# The Effectiveness of Distance Yoga Learning in Improving Maternal Pandemic-Related Depression and Stress During Pregnancy

**DOI:** 10.3390/healthcare13111345

**Published:** 2025-06-05

**Authors:** Wen-Ping Lee, Min-Yu Chang, Chiu-Tzu Lin, Whei-Mei Shih

**Affiliations:** 1 Department of Nursing, New Taipei Municipal TuCheng Hospital (Built and Operated by Chang Gung Medical Foundation), New Taipei City 236, Taiwan; pamela@cgmh.org.tw (W.-P.L.); yu@cgmh.org.tw (M.-Y.C.); q22208@cgmh.org.tw (C.-T.L.); 2 Department of Nursing, Chang Gung University of Science and Technology, Taoyuan 333, Taiwan; 3 Graduate Institute of Gerontology and Health Care Management, Chang Gung University of Science and Technology, Taoyuan 333, Taiwan

**Keywords:** distance yoga, pandemic, depression and stress, pregnancy

## Abstract

**Background:** The aim of this study was to explore the effects of distance yoga learning in improving depression and pregnancy stress in pregnant women during the pandemic. **Methods:** A preference-based quasi-experimental study design with an experimental group (*n* = 30) and a control group (*n* = 31) was used to test both self-reported depression and stress status. The experimental group received a 12-week pregnancy yoga program, including one weekly 60 min distance yoga class followed by two sessions of DVD yoga exercise at home, while the control group received only routine nursing care. **Results:** There were significant differences in the depression and pregnancy stress indices between the two groups. As the number of weeks of pregnancy increased, both pregnancy depression and stress tended to gradually increase (*p* < 0.001). However, the experimental group had less pregnancy depression than the control group (*p* < 0.001), while there was no significant difference between the two groups in stress (*p* = 0.970). **Conclusions:** The findings inform clinical practice regarding the use of alternative exercise options such as distance yoga classes for pregnant women’s mental health during a pandemic to reduce depression and pregnancy stress.

## 1. Introduction

According to the World Health Organization (WHO), the mortality rate after COVID-19 infection was higher among pregnant women than among non-pregnant women of the same age [1,2]. Pregnant women were 3 times more likely to require intensive care after infection, 2.9 times more likely to need a ventilator and 1.7 times more likely to die and had a significantly increased risk of severe illness and death [3]. Research showed that pregnant women infected with COVID-19 were more likely to suffer from premature birth, gestational hypertension, fetal growth restriction and other problems than those who were not infected. In addition, viral infections increased the need for cesarean sections and had potential health consequences for newborns, such as premature birth and low birth weight [4,5].

During the pandemic, loneliness and social isolation were publicly discussed. A systemic review showed that, on average, the quality of social relationships was perceived as worse during the pandemic than before [6]. Loneliness is one of the core indicators of well-being and is a robust predictor of depression, anxiety and suicidal ideation [7]. Due to a lack of information during COVID-19, people tended to have anxiety [8]. Pregnancy itself can be stressful, especially during a pandemic. A review of studies showed direct associations between maternal pandemic-related stress during pregnancy and infants’ temperament, socio-emotional development, and symptoms of anxiety and depression [9].

Postpartum depression (PPD) is a common and serious mental health problem that affects approximately 10% to 15% of women who have given birth. In addition to posing a threat to the mother’s own mental health, it may also interfere with the emotional connection and interaction quality between the mother and baby. Good mother–infant interaction plays a key role in the establishment of the infant’s early sociality, cognitive abilities and behavioral patterns. However, mothers with PPD often show a lower emotional sensitivity and expression of maternal love and tend to adopt more harsh or inconsistent parenting styles, and these parenting traits may further have a negative impact on children’s development [10,11].

Physical exercise is an important factor in maintaining the physical and mental health of pregnant women. Studies have pointed out that the psychological stress of pregnant women during the pandemic was significantly higher than that before the pandemic. This not only affected the health of the pregnant women themselves but also was related to premature birth, reduced mother–infant connection, postpartum depression and delayed cognitive/emotional development of infants [12,13,14].

Yoga, as an exercise that combines body, mind and breathing, has been proven to have many benefits for the physical and mental health of pregnant women. It can help pregnant women reduce psychological stress and anxiety, improve physical strength and flexibility, promote sleep quality, adjust postures, provide preparation for childbirth and enhance immunity [15]. Guidelines for practicing yoga are as follows: practice openly and in a peaceful environment, with an empty stomach, an empty bladder and bowel and a yoga mat [16]. Distance yoga learning allows pregnant women to practice at home, avoiding the close contact required to participate in physical classes. This approach may effectively reduce the risk of infection, safeguard the health of pregnant women and their fetuses, facilitate the practice of yoga in a familiar and comfortable environment, and minimize the inconvenience associated with going out [17].

People learned valuable experience from the challenges brought by the COVID-19 pandemic. Even though the COVID-19 pandemic may have passed, it seems that the world is woefully unprepared for the next. The WHO [18] calls on the world to strengthen preparation and response to potential epidemics and pandemics in the future. Countries around the world should formulate “epidemic treaties” to prepare for the next wave of “X disease”. While prenatal yoga is an effective way to improve mental health during pregnancy, and there is plenty of research on the stress-relieving benefits of yoga, few studies have evaluated its impact on mental health during times of extreme stress, such as a global pandemic. Therefore, the research hypothesis for this study was that yoga can reduce both depression and stress in pregnant women. The purpose of this study was to explore the impact of 12 weeks of distance yoga learning on depression and pregnancy stress in pregnant women during the COVID-19 pandemic.

## 2. Materials and Methods

This study employed a preference-based quasi-experimental study design for primiparous women in the obstetrics and gynecology department of a regional hospital as the research subjects. This trial followed the CONSORT 2010 checklist for reporting randomized controlled trials. This study received ethical approval from the hospital (IRB: 202200812B0), and all participants provided written informed consent. This protocol was registered on ClinicalTrials.gov with the registration number NCT06913582.

After the researcher took convenience samples and explained the research purpose in the prenatal parent classroom, participants were assigned to the experimental group and the control group according to the wishes of the cases. Pregnant women in the experimental group received routine prenatal care guidance and yoga exercises three times a week, while the control group only received routine prenatal guidance, and the differences between the two groups were compared.

The inclusion criteria included the following: 1. first-time mothers between 20 and 26 weeks of pregnancy; 2. singleton pregnancy with a normal fetus; 3. no history of smoking, drinking or drug abuse; 4. no bleeding during pregnancy; 5. no related high-risk complications during pregnancy (such as high blood pressure, preeclampsia, gestational diabetes, heart disease, etc.); 6. no early abortion symptoms; 7. able to move normally; 8. able to listen to, speak, read and write Chinese; and 9. no routine exercise behavior before. Exclusion criteria were as follows: 1. multiparous women; 2. first-time mothers with pregnancy > 26 weeks; 3. multiple births; 4. symptoms of bleeding or early miscarriage; or 5. related high-risk pregnancy complications.

The sample size was estimated using G-power 3.1.9.2 software. According to statistical standards, the α value was set to 0.05, and the 1-β value was 0.80. Referring to the research conducted by Newham [19], effect size = 0.70 was set, and the sample size was estimated to be 52 participants. Assuming the attrition rate was 20%, there were 32 people in the experimental group and the control group, making a total of 64 participants.

**Figure 1:**
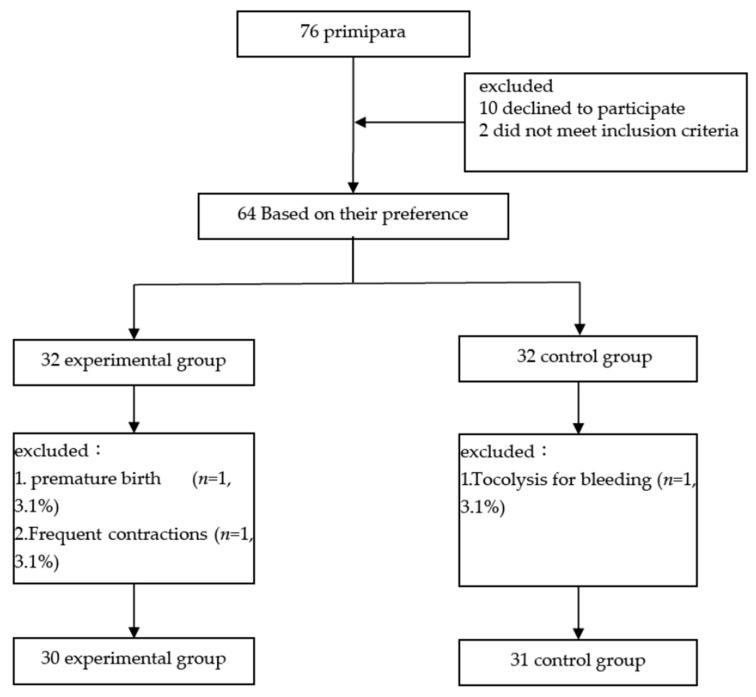
Flow chart of data collection.

From 23 June 2022 to 20 June 2023, a total of 76 primiparous women were contacted, and 64 were willing to participate. They were divided into experimental groups and control groups according to the mothers’ wishes. During this study, one participant in the experimental group had a premature delivery due to early water rupture, and the other one was hospitalized for tocolysis due to frequent uterine contractions and therefore withdrew from the experiment, resulting in the loss of two samples. For the control group, one participant was admitted to the hospital for tocolysis due to prepartum hemorrhage and was withdrawn from this study. Therefore, a total of 61 participants completed this study (30 in the experimental group and 31 in the control group) (Figure 1).

### 2.1. Data Collection Tool

Pregnant women in this study were asked to fill out the self-reported questionnaires including basic characteristics, the Edinburgh Postnatal Depression Scale (EPDS) and the Pregnancy Stress Rating Scale (PSRS).

(1) Basic: Basic personal information included age, height, religious beliefs, marital status, education, daily activities, past medical history, etc. Obstetric variable information included weeks of pregnancy, pre-pregnancy weight, weight gained during pregnancy, exercise before and during pregnancy, etc.
(2) Edinburgh Postnatal Depression Scale (EPDS): The Edinburgh Postnatal Depression Scale is used to measure prenatal and postpartum depressed mood [20]. There are 10 questions in total, each question is scored on a 4-point scale. According to the order of each option, the first, second and fourth items are scored from 0 to 3 points, the other items are scored from 3 to 0 points, and the total score is from 0 to 30 points. The levels of scale were as follows: 0–9 points: low risk, no further psychological intervention is usually needed; 10–12 points: medium risk, further evaluation and observation are recommended; 13 points and above: high risk, comprehensive psychological evaluation and possible intervention measures should be carried out. The higher the score of pregnant women, the more severe the prenatal depression is. The internal consistency coefficient was 0.87 [21].
(3) Pregnancy Stress Rating Scale (PSRS): The “Pregnancy Stress Scale” developed by Chen, Huang and Ka in 1991 and revised into a new version of the “Pregnancy Stress Scale” by Chen [22] was used in this study. The scale has five factors; it mainly measures the psychological stress experienced by women during pregnancy. There are 36 questions in total, and the Likert five-point scoring method is used, resulting in a maximum total score of 180 points. The higher the score, the greater the stress during pregnancy. The new version of the “Pregnancy Stress Scale” has good internal consistency (α = 0.92) and 2-week test–retest reliability (α = 0.82).

### 2.2. Program Design

A 12-week pregnancy yoga program was delivered to the experimental group (*n* = 30), including one weekly 60 min remote online yoga class followed by two sessions of DVD yoga exercises. The control group (*n* = 31) was given general routine nursing guidance at 20–24 weeks of pregnancy.

The content of the course was based on the yoga exercise DVD for use during pregnancy jointly developed by the medical team and professional yoga teachers and is shown in Table 1. Each class was taught remotely by a certified professional pregnancy yoga teacher (the rest of the classes were performed at home according to the exercise DVD, twice a week, 60 min/time). At the beginning of the class, the yoga teacher asked the participants to relax, used soothing music to relax the body, and included breathing methods, body postures (including sitting warm-ups, four-legged kneeling posture changes, standing postures, supine postures, holding on to walls and chairs, leaning against walls, etc.), pelvic floor muscle training and practices such as meditation and relaxation. After the course, participants were asked to practice with the yoga exercise DVD at home, twice a week, and to record their exercise status. The researcher accompanied the distance course throughout the entire process to understand implementation issues and provided consultation via a telephone interview each week. The participants in the experimental group responded with 100% adherence.

### 2.3. Statistical Analysis

Data were analyzed using SPSS version 25 for descriptive statistics such as percentage, mean and standard deviation and inferential statistics such as *t*-test, ANOVA and generalized estimating equation (GEE).

## 3. Results

In order to ensure the compliance of the experimental group, the researchers called each participant every week according to the attendance list, firstly to remind them and secondly to record 100% completion. The basic attributes of the participants are shown in Table 2. The average age was 32.25 years old (SD = 3.846), the average height was 160.75 cm (SD = 4.703) and the average pre-pregnancy weight was 55.64 kg (SD = 6.802). Most participants had college or below education (*n* = 51, 83.6%) and were married, with 58 married people accounting for 95.1% of all participants. As for religious belief, Taoism had the largest number of people, 26 (42.6%). In terms of daily activities, 58 people (95.1%) walked between 1 and 3 h a day. There was no statistically significant difference in all variables (*p* > 0.05), indicating the homogeneity of the two groups at the baseline.

### 3.1. The Change in Depression Status

During the 20th to 26th weeks of pregnancy, the overall average score was 11.25, indicating a medium risk. The average score was 11.29 for the control group and 11.20 for the experimental group. From 24 weeks to 30 weeks of pregnancy, the overall average total score was 12.64, which was classified as high risk, with the average total score of the control group being 14.77 and the experimental group being 10.50. From 28 weeks to 34 weeks of pregnancy, the overall average total score was 11.96, which was classified as medium risk, with the average total score of the control group being 15.94 and the experimental group being 7.97. From 32 weeks to 38 weeks of pregnancy, the overall average total score was 12.32, and the average total score was 17.61 in the control group and 7.03 in the experimental group. The results of two independent t-tests indicated that the impact of depression during pregnancy on women in the experimental group was significantly lower than in the control group at 24–30 weeks (*t* = 5.608, *p* < 0.001), 28–34 weeks (*t* = 10.651, *p* < 0.001), and 32–38 weeks (*t* = 15.776, *p* < 0.001) of pregnancy, with all differences reaching statistical significance (Table 3).

### 3.2. Stress During Pregnancy

During the 20th to 26th weeks of pregnancy, the overall average total score was 92.99 points, the average total score of the control group was 94.45 points and the average total score of the experimental group was 91.53 points. From 24 weeks to 30 weeks of pregnancy, the overall average total score was 107.99 points, the average total score of the control group was 110.65 points and the average total score of the experimental group was 105.33 points. From 28 weeks to 34 weeks of pregnancy, the overall average total score was 121.26 points, with the average total score of the control group being 124.19 points and the experimental group being 118.33 points. During the 32nd to 38th week of pregnancy, the overall average total score was 127.84 points, with the average total score of the control group being 129.35 points and the experimental group being 126.33 points. The independent *t*-test results of the two groups showed that the impact of stress during pregnancy on pregnant women in the experimental group from 24 weeks to 30 weeks of pregnancy (*t* = 2.075, *p* < 0.05) and from 28 weeks to 34 weeks of pregnancy (*t* = 3.361, *p* = 0.001) was significantly lower than that of the control group and reached a statistically significant difference. Although the experimental group was less affected by stressful situations than the control group from 32 to 38 weeks of pregnancy, there was no statistically significant difference (*p* > 0.05) (Table 4).

### 3.3. The Association of Pregnant Women’s Yoga Exercises Using Generalized Estimation Functions

**Figure 2:**
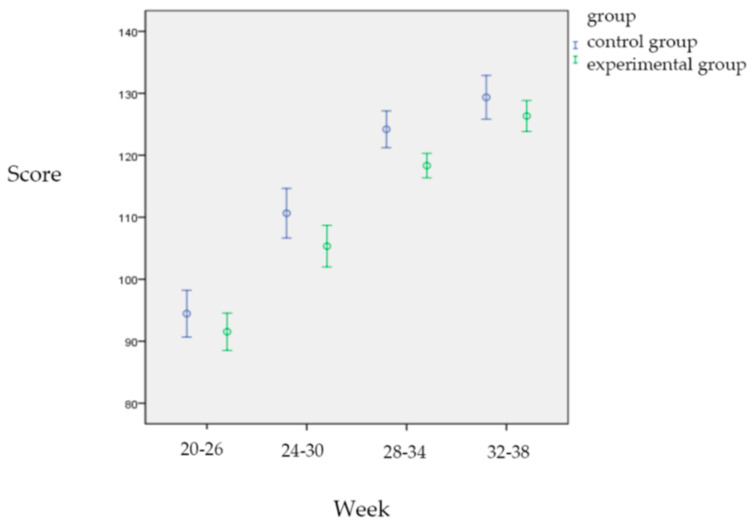
Comparison of Edinburg depression scores between two groups.

In terms of depression, the control group showed a gradual increase in depression as the number of weeks of pregnancy increased, while there was no significant difference in the experimental group. After 12 weeks of pregnancy yoga, the experimental group had 0.090 points less discomfort during pregnancy than the control group, which did not reach a statistically significant difference (*p* = 0.796). As the number of weeks of pregnancy increases, pregnancy depression tended to gradually increase (*p* < 0.001); however, the results of cross-comparison between time and group showed that the experimental group had 10.489 points less pregnancy depression at 32 to 38 weeks than at 20 to 26 weeks of pregnancy (*p* < 0.001), but depression tended to increase gradually with the increase in pregnancy weeks in the control group (Figure 2, Table 5).

**Figure 3:**
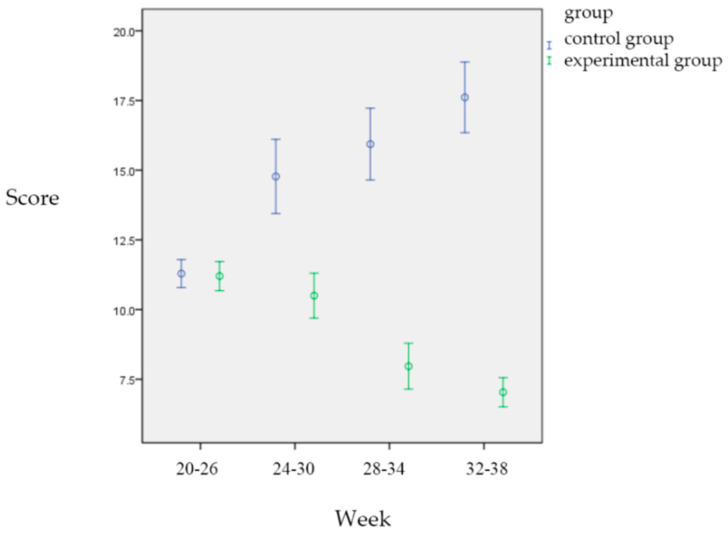
Comparison of stress between the two groups during different pregnancy periods.

Figure 3 shows that as the number of weeks of pregnancy increased, the stress in the control group gradually increases, while there was no significant difference in the experimental group. After 12 weeks of yoga, although the experimental group had 2.918 points less discomfort during pregnancy than the control group, this did not reach a statistically significant difference (*p* = 0.210). As the number of weeks of pregnancy increased, the stress of pregnancy tended to gradually increased (*p* < 0.001). However, the results of cross-comparison between elapsed time and group showed that the experimental group’s stress at 32 to 38 weeks was 0.103 points less than that at 20 to 26 weeks of pregnancy (*p* = 0.970), which means that the stress of the experimental group was compared with the control group. There was no significant difference between the two groups. The stress of both groups showed a gradual increase with the increase in pregnancy weeks. However, the results of cross-comparison between time and group showed that the experimental group’s stress at 32 to 38 weeks was 0.103 points less than that at 20 to 26 weeks of pregnancy (*p* = 0.970), indicating that the stress of the experimental group was not significantly different from that of the control group. The stress of both groups showed a tendency to gradually increase with the increase in pregnancy weeks (Figure 3, Table 6).

## 4. Discussion

Overall, the study participants’ activities/exercises before pregnancy and during pregnancy were the highest in those who were active for 1–3 h a day, while those who did not exercise regularly in the year before pregnancy were the most common. This is consistent with the findings of scholars who have proposed pregnant women do not exercise regularly during pregnancy [23,24]. The study by Walasik [25] also found that pregnant women exercised less. Most late-term pregnancies involve mainly walking-based exercise. This result is consistent with the findings of Lee [23]. According to Chinese customs, the older generation always believed that it was best not to “move” rashly during pregnancy. Pregnant women were expected to keep quiet and be particularly careful, even with simple housework. They were expected to move less, take more supplements and rest more. This is consistent with the fact that pregnant women were prone to discomfort during pregnancy due to lack of exercise.

The absence of a significant difference in stress levels between the two groups may be attributed to the fact that all pregnant women were concerned about peripartum safety. Although the experimental group experienced a significant reduction in stress following the 12-week intervention, when the data from the control group were included in the Generalized Estimating Equation (GEE) analysis (Group × Time interaction), the overall stress scores did not show a statistically significant difference. This implies that yoga can be an effective exercise for pregnant women, as the control group had a higher stress score, diluting the statistical significance of yoga exercise. The research results showed that a 12-week prenatal yoga program can improve prenatal depression. The experimental group’s depression level was lower than that of the control group, and it was statistically significantly lower than that of the control group from 24 weeks to 38 weeks of pregnancy. The results of this study were consistent with most prenatal yoga studies, which agreed that prenatal yoga had benefits for both short- and long-term mental health improvements [26,27,28]. However, this study’s impact of a prenatal tele-yoga intervention on pregnant women during times of extreme stress, such as a global pandemic, was clinically important. Because pregnant women were particularly stressed during the pandemic [29], their stress and depression could have had a serious impact on maternal and infant health [30]. Studies have shown that prenatal yoga significantly improved maternal physical health parameters, including blood pressure and IUGR, and mental health during pregnancy [5,31]; therefore, prenatal yoga improved birth outcomes by affecting mental health.

The literature suggests that during the pandemic, lifestyle factors like sleep, social interaction and physical activity had a stronger association with depression compared to the period before the pandemic [31]. Yoga had a positive effect on improving depression outcomes, with a standardized mean difference of −0.41 (95% CI −0.65 to −0.17) [32]. Wu [14] compared the mental health outcomes of 4124 pregnant women from 10 different provinces in China before and after COVID-19 and found that the prevalence of depressive symptoms was higher among pregnant women (29.6% vs. 26.0%), an increase of 3.4% that reached statistical significance (*p* = 0.2). There was a positive correlation between the prevalence of depression and the daily number of new confirmed COVID-19 cases, suspected infections and deaths. Davenport [13] also found that psychological distress among pregnant women increased during COVID-19. An online survey of 900 Canadian women (520 pregnant women) showed that the prevalence of depression and anxiety more than doubled; 15% of respondents suffered from depression before the pandemic, while during the pandemic, the proportion of depression increased to 40.7%. Ceulemans [33] conducted an online survey of 5866 women (2421 pregnant women) in Belgium and found that nearly half of the women experienced symptoms of depression or anxiety during the pandemic lockdown. The prevalence of severe depression symptoms was 25.3%, of which 14% met the criteria for severe depressive symptoms.

Additionally, this study suggests that longer yoga interventions (more than eight weeks) may be more effective in achieving significant improvements in mental health, aligning with findings from previous research. Davis [34] examined 46 pregnant women experiencing symptoms of depression and anxiety. After eight weeks of prenatal yoga, no significant differences were observed in anxiety and depression levels between the yoga group and the control group. Newham [19] conducted a study on 59 first-time mothers and found no significant difference in anxiety levels between the yoga group and the control group after eight weeks of prenatal yoga. Duchette [35] found that a 10-week prenatal yoga program significantly improved mental health, with notable reductions in anxiety (*p* = 0.002), depression (*p* = 0.032) and overall mood disorders (*p* = 0.002) in the yoga group. Field [36] demonstrated that a 12-week prenatal yoga and massage intervention led to greater reductions in anxiety and depression compared to the control group. This study significantly contributes to the field by highlighting the supportive association of prenatal yoga with maternal mental health during the COVID-19 pandemic.

Throughout this study, the same experienced and qualified prenatal yoga instructor led all sessions. Her extensive teaching background fostered trust among participants, encouraging them to share their pregnancy experiences in class. Participants had the option to attend as avatars or with their cameras on, giving them autonomy over their participation format. This flexibility allowed them to choose the environment where they felt safest, enhanced adherence to the practice and removed the stress of commuting to a yoga studio. Lastly, although this study took place during a pandemic, it provided a unique opportunity to examine mental well-being and assess the impact of prenatal yoga.

## 5. Conclusions

This study examined the impact of distance yoga exercise on depression and pregnancy stress in pregnant women during the COVID-19 pandemic. However, although data collection occurred during the pandemic period, the findings have direct clinical implications for women who are pregnant during a pandemic, benefiting the mental health of expectant mothers. Given the potential for future pandemics and the unpredictable nature of such crises, maternal mental health plays a vital role in both maternal and fetal well-being. Therefore, it is essential to prioritize and support the mental health of pregnant women during pandemic conditions. After a 12-week remote yoga intervention, participants reported significantly lower levels of depression and pregnancy-related stress compared to the control group. This suggests that prenatal yoga may be a viable and more widely accepted alternative to standard treatments for prenatal depression. Therefore, healthcare providers may recommend distance yoga to pregnant women, in addition to other lifestyle changes, as an alternative way to improve physical and mental health and exercise in the future when facing a pandemic.

## 6. Limitations

The assignment of participants to groups was based on their preference for most of their requests, which introduces a risk of selection bias. The experimental group potentially had a stronger intention to exercise than the control group, which would affect the outcome. Since this was a preference-based quasi-experiment and only one researcher implemented the program, it was difficult to blind the outcome assessment. In addition, the relatively small number of participants limits the generalizability of the findings. It is recommended that randomization and larger sample sizes be implemented in future studies for more precise findings. The questionnaires used are self-reported and thus have limitations due to potential reporting bias.

## Figures and Tables

**Table 1:** Contents of yoga exercise.

Activity and Posture	Time
**Warm-up**	10 min
**Relax and feel every part of body**	5 min
**Adjust breathing and awareness**	5 min
**Posture practice**	40 min
**Sukhasana side stretch (easy seated side stretch)**	3–5 breaths/each side
**Marjaryasana (cat pose)**	5 breaths
**Child’s pose (child’s pose)**	5 breaths
**Parighasana (door pose)**	3–5 breaths/each side
**Anjaneyasana (crescent moon pose)**	3–5 breaths/each side
**Tadasana (mountain pose)**	5 breaths
**Ardho mukhasana (downward dog pose)**	3–5 cycles
**Trikonasana (triangle pose)**	3–5 breaths/each side
**Vrikshasana (tree pose)**	3–5 breaths/each side
**Badakonasana (binding angle pose)**	5 breaths
**Meditation**	10 min
**Meditation, relax and focus**	10 min

**Table 2:** Comparison of demographic data and exercise history of the two groups (N = 61).

Item	Experiment (N = 30)		Control (N = 31)		*χ* ^2^ */t*	*p*
	*n*	%	*n*	%		
Age	30	32.63 ± 3.11	31	31.87 ± 4.46	0.771 ^B^	0.441
Height	30	160.3 ± 3.91	31	161.19 ± 5.39	−0.739 ^B^	0.463
Weight before pregnancy	30	57.7 ± 7.25	31	53.65 ± 5.78	2.420 ^B^	0.069
Education					0.403 ^A^	0.525
college and below	26	86.7	25	80.6		
master’s and above	4	13.3	6	19.4		
Marriage					0.317 ^A^	0.573
married	29	96.7	29	93.5		
single	1	3.3	2	6.5		
Religion					1.842 ^A^	0.398
none	12	40.0	12	38.7		
Buddhism	7	23.3	4	12.9		
Taoism	11	36.7	15	48.4		
Job					0.851 ^A^	0.837
service industry	24	80.0	24	77.4		
government	3	10.0	2	6.5		
medical	1	3.3	1	3.2		
industry and commerce	2	6.7	4	12.9		
Disease					0.386 ^A^	0.534
none	28	93.3	30	96.8		
yes (thyroid)	2	6.7	1	3.2		
Daily activity					0.386 ^A^	0.534
walking 1–3 h/day	28	93.3	30	96.8		
walking 4–6 h/day	2	6.7	1	3.2		

Note: ^A^ is the chi-square test; ^B^ is the independent *t*-test.

**Table 3:** Comparison of demographic data and depression of the two groups (N = 61).

Item	Experiment		Control		*t*	*p*
	M	SD	M	SD		
Total scores						
20–26 weeks	11.20	1.400	11.29	1.371	0.255	0.800
24–30 weeks	10.50	2.162	14.77	3.631	5.608	<0.001 ***
28–34 weeks	7.97	2.205	15.94	3.511	10.651	<0.001 ***
32–38 weeks	7.03	1.402	17.61	3.451	15.776	<0.001 ***

Note: *** *p* < 0.001.

**Table 4:** Comparison of demographic data and stress of the two groups (N = 61).

Item	Experiment		Control		*t*	*p*
	M	SD	M	SD		
Total scores						
20–26 weeks	91.53	8.085	94.45	10.311	1.227	0.225
24–30 weeks	105.33	8.996	110.65	10.935	2.075	0.042 *
28–34 weeks	118.33	5.307	124.19	8.072	3.361	0.001 **
32–38 weeks	126.33	6.687	129.35	9.639	1.426	0.160

Note: * *p* < 0.05; ** *p* < 0.01.

**Table 5:** Comparison of depression in Edinburgh during pregnancy between the experimental group and the control group using a generalized estimation function.

Item	*B*	*SE*	95% Confidence Interval		Wald *χ*^2^	*p*
			Lower Bound	Upper Bound		
Group						
Experiment	−0.090	0.3490	−0.774	0.594	0.067	0.796
Control	Reference					
Time						
32–38 weeks	6.323	0.6757	4.998	7.647	87.553	<0.001 ***
28–34 weeks	4.645	0.6682	3.335	5.955	48.325	<0.001 ***
24–30 weeks	3.484	0.6962	2.119	4.848	25.044	<0.001 ***
20–26 weeks	Reference					
Group × Time						
Group × 32–38 weeks	−10.489	0.7296	−11.919	−9.059	206.688	<0.001 ***
Group × 28–34 weeks	−7.878	0.7654	−9.379	−6.378	105.958	<0.001 ***
Group × 24–30 weeks	−4.184	0.7412	−5.637	−2.731	31.860	<0.001 ***
Group × 20–26 weeks	Reference					

Note: Group: Experiment vs. Control; Time: pregnancy weeks; *** *p* < 0.001.

**Table 6:** Comparison of stress during pregnancy between the experimental group and the control group using a generalized estimation function.

Item	*B*	*SE*	95% Confidence Interval		Wald *χ*^2^	*p*
			Lower Bound	Upper Bound		
Group						
Experiment	−2.918	2.3292	−7.483	1.647	1.570	0.210
Control	Reference					
Time						
32–38 weeks	34.903	2.0803	30.826	38.980	281.511	<0.001 ***
28–34 weeks	29.742	1.7966	26.221	33.263	274.050	<0.001 ***
24–30 weeks	16.194	1.8233	12.620	19.767	78.878	<0.001 ***
20–26 weeks	Reference					
Group × Time						
Group × 32–38 weeks	−0.103	2.7507	−5.494	5.288	0.001	0.970
Group × 28–34 weeks	−2.942	2.4184	−7.682	1.798	1.480	0.224
Group × 24–30 weeks	−2.394	2.4324	−7.161	2.374	0.968	0.325
Group × 20–26 weeks	Reference					

Note: Group: experiment vs. control; Time: pregnancy weeks; *** *p* < 0.001.

## Data Availability

The datasets used and/or analyzed during the current study are available from the corresponding author upon reasonable request.
